# Lifestyle triggers of migraine: Sleep restriction and caffeine lower the threshold for migraine‐like responses in rats in a sex‐specific manner

**DOI:** 10.1111/head.70099

**Published:** 2026-04-09

**Authors:** Gabriel Camargo de Oliveira, Darciane Favero Baggio, Vanessa Bordenowsky Pereira Lejeune, Laura Storithont Quinelato, Fernanda Mariano Ribeiro da Luz, Aleksander Roberto Zampronio, Luana Fischer, Juliana Geremias Chichorro

**Affiliations:** ^1^ Department of Pharmacology Federal University of Paraná Curitiba Brazil; ^2^ Department of Physiology Federal University of Paraná Curitiba Brazil

**Keywords:** caffeine, calcitonin gene‐related peptide, mechanical allodynia, photosensitivity, pituitary adenylate cyclase‐activating polypeptide, sleep restriction

## Abstract

**Objective:**

This study explores whether sleep restriction (SR) and caffeine intake affect migraine susceptibility by testing if each condition, alone or in combination, precipitates migraine‐like responses to subthreshold doses of calcitonin gene‐related peptide (CGRP) or pituitary adenylate cyclase‐activating polypeptide (PACAP) in male and female rats.

**Background:**

Migraine is a debilitating neurological syndrome that affects approximately 15% of the global population, with a three‐fold higher prevalence in females compared to males. Among the peripheral mechanisms underlying migraine, the release of vasoactive peptides by trigeminal ganglion (TG) neurons, such as CGRP and PACAP, plays a crucial role. Various environmental triggers—including sleep or food deprivation, caffeine intake or withdrawal, stress, and light exposure—have been associated with the onset of migraine attacks; however, the mechanisms by which these factors modulate nociceptive sensitization remain poorly understood.

**Methods:**

Male and female Wistar rats were subjected to SR for 6 h daily over 3 consecutive days using the gentle handling method, and the periorbital mechanical allodynia was assessed using von Frey filaments before and after each day of SR. Next, a subthreshold dose of CGRP (38 ng/10 μL) or PACAP (0.1 ng/10 μL) was administered into the TG on the third day of SR to evaluate whether sleep loss enhances susceptibility to migraine‐like responses. Finally, two additional experiments were conducted to investigate the influence of caffeine (50 mg/kg, orally) exposure in combination of SR in CGRP and PACAP effects. In all experiments, on day 4 (i.e., 24 h after the last SR), the animals were exposed for 1 h to an aversive light for verification of latent sensitization.

**Results:**

The results demonstrated that SR alone did not alter the periorbital mechanical threshold in either male or female rats. However, when SR was combined with the administration of CGRP or PACAP at subthreshold doses, a significant periorbital mechanical allodynia developed in female, but not in male rats. The exposure to light in the subsequent day caused a transitory reactivation of mechanical allodynia only in females. In well‐rested animals, a 3‐day caffeine regimen enabled behaviorally subthreshold doses of CGRP or PACAP to elicit migraine‐like responses in females, but not in males. In sleep‐restricted animals, combining caffeine with subthreshold doses of CGRP or PACAP rendered males susceptible to migraine‐like responses and markedly exacerbated these responses in females, including 1 day later, after light exposure.

**Conclusion:**

These findings suggest that SR facilitates trigeminovascular sensitization, promoting migraine‐like responses in a sex‐specific manner and highlighting caffeine as an enhancer of this interaction. Beyond reinforcing the association between poor sleep and migraine, the data offer new insights into the involvement of the purinergic system and sex differences in migraine pathophysiology.

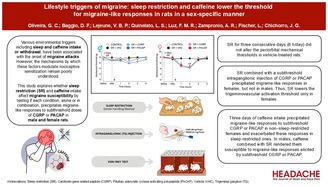

## Plain Language Summary

Sleep restriction increases sensitivity to migraine triggers. In this study, rats exposed to sleep restriction and caffeine showed more intense and longer‐lasting pain responses after receiving migraine‐inducing peptides, with female animals showing the strongest effects. These findings suggest that poor sleep and caffeine use together may worsen migraine attacks, especially in female patients.

## Plain Language Summary

Sleep restriction (SR) and caffeine intake act as environmental modulators of migraine susceptibility. Their effects are sex‐dependent: Females show heightened sensitivity under both SR and caffeine conditions. Males require the combination of SR and caffeine to exhibit susceptibility.

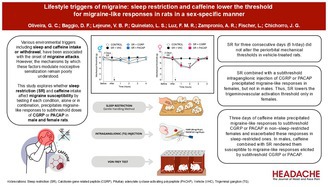

**Abbreviations**
CGRP: calcitonin gene‐related peptide
PACAP: pituitary adenylate cyclase‐activating polypeptide
SR: sleep restriction
TG: trigeminal ganglion

## INTRODUCTION

Migraine is a neurovascular disorder that affects approximately 15% of the global population, making it one of the most prevalent neurological conditions worldwide, being two to three times more prevalent in females.^1^ Central to its pathophysiology is the trigeminovascular system, where activation of meningeal trigeminal nociceptors triggers the release of vasoactive neuropeptides, most notably calcitonin gene‐related peptide (CGRP), which in turn promotes vasodilation and both peripheral and central sensitization.^2^ More recently, pituitary adenylate cyclase‐activating polypeptide (PACAP) has emerged as a parallel effector, inducing mast cell degranulation and nociceptor sensitization. These pathways converge in a feed‐forward loop of neurogenic inflammation.^2^, ^3^ Building on these mechanistic insights, recent advances in migraine therapy have centered on monoclonal antibodies targeting CGRP—now in clinical use—and PACAP, which is currently undergoing phase 2 trials.^3^

Consistent with this pathophysiological framework, rodent models employing neuropeptide‐based paradigms have demonstrated strong translational relevance. Recognition that monoclonal antibodies targeting CGRP exhibit minimal penetration of the blood–brain barrier^2^, ^4^ has shifted focus toward peripheral migraine models, with direct injection of these neuropeptides into the trigeminal ganglion (TG) emerging as particularly advantageous.^5^, ^6^ This approach avoids systemic cardiovascular artifacts that can confound behavioral readouts^7^ and enhances the TG's neuron–glia crosstalk, driving ganglion‐originated peripheral and central sensitization.^5^, ^6^

Poor sleep, whether from clinical sleep disorders or social demands, has become increasingly common worldwide, with estimated rates ranging from 20% to 70% of the general population.^8^ Epidemiological studies reveal a bidirectional link between migraine and sleep: patients with migraine are more prone to sleep restriction (SR), whereas insufficient sleep increases the risk of migraine onset, chronification, and attack frequency.^9^ Despite this strong association, the underlying mechanisms remain largely unexplored, underscoring the need for targeted animal models. One study showed that 6 h of acute SR sensitizes female mice to subthreshold dural CGRP,^10^ but it remains unknown whether prolonged SR alters susceptibility to migraine‐like responses induced by CGRP or PACAP injected into the TG. Caffeine is one of the most widely consumed psychoactive substances worldwide, with approximately 80% of the global population ingesting at least one caffeinated beverage daily.^11^ Clinical data reveal a complex, bidirectional role of caffeine in migraine: although withdrawal can trigger migraine attacks,^12^ its combination with analgesics significantly enhanced treatment efficacy.^13^ However, although caffeine consumption is elevated among individuals with poor sleep, its effects on migraine under sleep‐deprived conditions remain unexplored.

Therefore, in this study, we investigated whether SR and caffeine intake interact to influence migraine‐like pain. First, we tested whether 3 days of SR (6 h per day) precipitate migraine‐like responses in male and female rats receiving subthreshold doses of CGRP or PACAP into the TG. Then, we examined the impact of 3 days of caffeine intake on the development of migraine‐like responses in both sleep‐restricted and well‐rested animals.

## MATERIALS AND METHODS

### Animals

A total of 320 male and female adult Wistar rats, weighing 220–300 g, were used in this study. The animals were housed in the same animal facility, separated by sex in plastic cages (four per cage) containing wood shavings, in an enriched environment with controlled light (12‐h light/dark cycles; 7:00 a.m. to 7:00 p.m.) and with food and water ad libitum. All experimental protocols were performed during the light phase, separately for each sex. All procedures were conducted in accordance with ARRIVE guidelines and were previously approved by the UFPR Ethics Committee (CEUA‐BIO‐UFPR #1507). The animals were assigned to the different experimental groups by sortition, and the sample size was determined based on the G*Power 3.1 software, defining a large standardized effect of *F* = 0.5; power of 0.85 and *α* = 0.05, and estimated 10 rats per group.

### Drugs

Rat alpha isoform of calcitonin gene‐related peptide (α‐CGRP) and pituitary adenylate cyclase‐activating polypeptide‐38 (PACAP‐38) were obtained from Sigma‐Aldrich (St. Louis, MO, USA). Both were dissolved in 0.9% sodium chloride at concentrations of 38 ng/10 μL and 0.1 ng/10 μL, respectively, immediately before the intraganglionic injection. Both neuropeptides were administered at subthreshold doses (i.e., doses that do not induce behavioral responses), corresponding to one‐tenth of the doses previously reported to induce periorbital mechanical allodynia, as established in prior studies.^5^, ^6^

Caffeine (Vetec, SP, Brazil) was diluted in 0.9% sodium chloride to a concentration of 25 mg/mL immediately before the administration at a dose of 50 mg/kg.^14^, ^15^, ^16^ The oral bioavailability of caffeine in rats is approximately 90% and there is no difference in the pharmacokinetics and behavioral effects compared to the intraperitoneal route. These observations justify the administration by gavage, which is less invasive than the intraperitoneal route for repeated treatments. Halothane (Tanohalo, Cristalia, Brazil) was used for inhalation anesthesia.

### Intraganglionic injection

The injection was performed according to previous studies.^5^, ^6^ After brief inhalation anesthesia with halothane (approximately 3 min), the animal's head was restrained, and a long sterile 27‐G needle connected to a 0.5 mL Hamilton syringe was inserted and positioned at a 10° angle to the midline in the zygomatic process. The needle was inserted through the infraorbital canal until its tip passed the foramen rotundum and reached the TG. Only the right TG was injected with CGRP, PACAP, or vehicle and the injection volume was 10 μL. After the injection (performed over approximately 2 min), animals were monitored until complete recovery from anesthesia (approximately 5 min).

### SR

The rats were subjected to SR for a period of 6 h (from 7:00 a.m. until 1:00 p.m.) daily for 3 days using the enriched gentle handling method.^17^, ^18^ Briefly, the method consists of using gentle manipulation to prevent the animal from settling down to sleep. To keep the animals awake, they were gently touched with a soft‐bristled brush. The experimenter introduced new small objects into the environment and, if necessary, gently moved the home cage in a seesaw motion. The control groups were kept in the same conditions but were allowed to sleep freely.

### Assessment of periorbital mechanical allodynia

The animals were placed individually in 30 cm^3^ acrylic observation boxes for at least 1 h for habituation. The mechanical threshold of the periorbital region was assessed with von Frey filaments (Semmes‐Weinstein monofilaments, Stoelting, Wood Dale, IL, USA), applied to the midline of the forehead. Eight filaments, from 0.04 to 8.0 g, were applied in ascending order, three times each, respecting an interval of 30 s between each application. The filament that evoked two positive behaviors such as attack, escape, and/or rapid withdrawal of the head was considered the periorbital mechanical threshold.^5^, ^6^ Only rats showing baseline responses equal to 8 g were included in the experiments. During this test, the experimenters were blinded to the animals' condition.

### Photosensitivity assessment

The photosensitivity protocol was performed based on previously published studies,^5^, ^6^ with animals kept in their home cage and exposed to 5000 lux of illumination for a period of 1 h, using a 180‐watt lamp, with the room lighting during the experiment being 0 to 1 lux. Periorbital cutaneous allodynia was assessed every hour for up to 4 h following light exposure and, when indicated by the persistence of the behavioral response, additionally at 24 and 48 h.

### Experimental design

**FIGURE 1:**
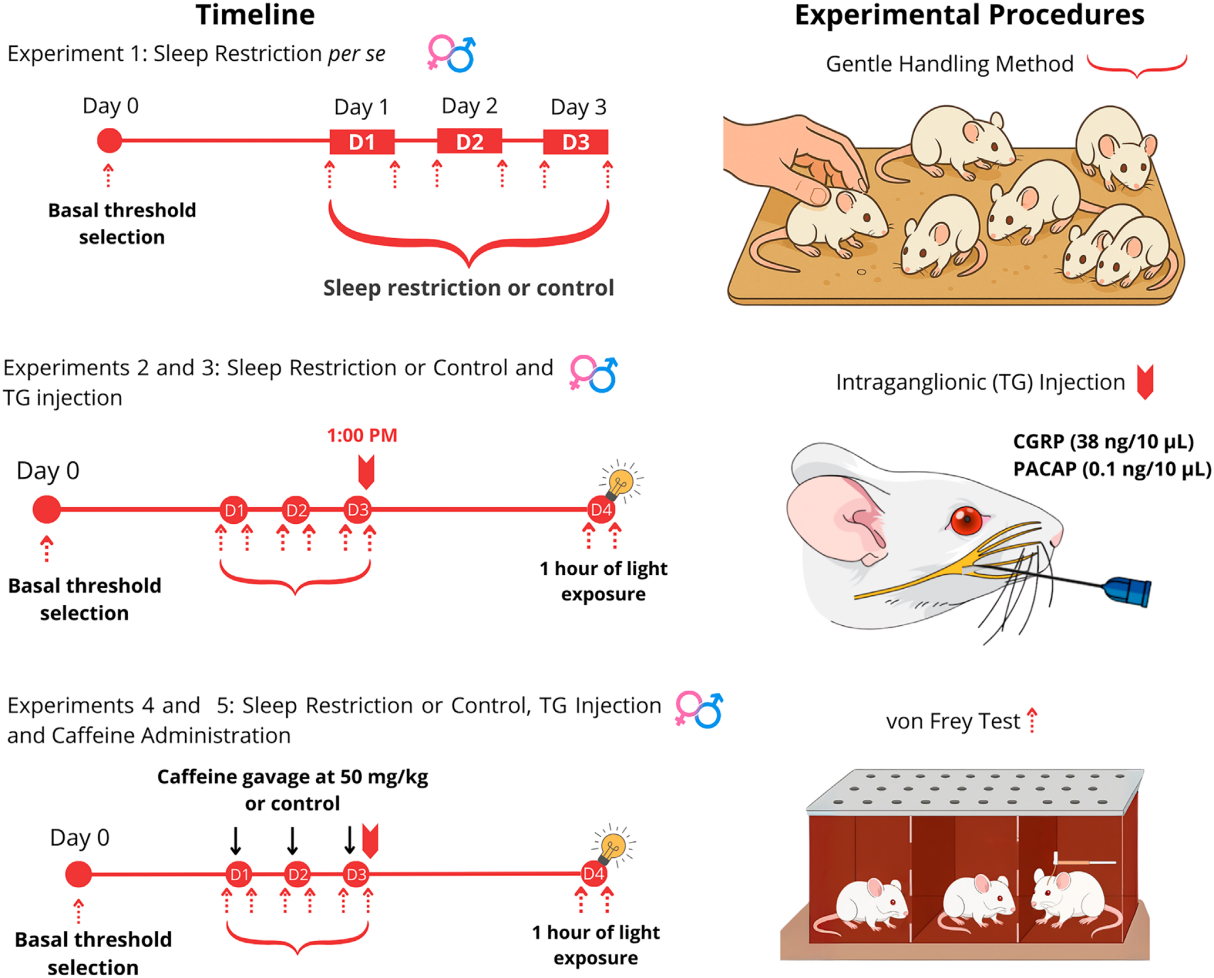
The timeline of experiments 1–5 is depicted on the left column and the experimental procedures are illustrated on the right column. CGRP, calcitonin gene‐related peptide; PACAP, pituitary adenylate cyclase‐activating polypeptide. Created with Biorender. [Color figure can be viewed at wileyonlinelibrary.com]

The experimental procedures are represented in Figure 1.

Experiment 1 was performed to determine whether SR per se would influence the periorbital mechanical threshold of male and female rats. The baseline periorbital mechanical threshold of animals was assessed followed by the SR protocol (i.e., 6 h of SR). At the end of the SR, the von Frey test was repeated each day.

In experiments 2 and 3, male and female rats were subject to the same procedures described in experiment 1. However, on day 3, independent groups of rats received an intraganglionic injection of CGRP or vehicle (experiment 2) or PACAP or vehicle (experiment 3) at 1 p.m. At the end of the SR period on day 3, the von Frey test was performed at 1‐h intervals up to 4 h after the injection. On day 4, the rats were exposed to the aversive light for 1 h, and the von Frey test was performed before exposure to light, and at 1‐h intervals up to 4 h.

Experiments 4 and 5 were performed to test the hypothesis that caffeine would counteract the effect of SR. The control group received caffeine orally for 3 days, and on day 3 at 1 p.m., received an intraganglionic injection of CGRP, PACAP, or the vehicle, but was not subjected to SR. All additional experimental groups were subjected to SR and received the following treatments: vehicle or caffeine, orally for 3 days, before the SR period; CGRP, PACAP, or vehicle, intraganglionic, on day 3 at 1 p.m., immediately after the last SR period. The von Frey test was performed on days 1, 2, and 3, before and after the SR period, and on day 3 also at 1‐h intervals up to 4 h after the intraganglionic treatments. On day 4, the animals were exposed to the aversive light for 1 h, and the von Frey test was performed before exposure to light, at 1‐h intervals up to 4 h and again at 24 and 48 h after light exposure.

### Statistical analysis

Data were expressed as mean ± standard error of the mean (SEM). Two‐way repeated measures analysis of variance (ANOVA), with treatment and time as independent factors, followed by Bonferroni's post hoc test, was used to analyze the time course of periorbital mechanical allodynia and photosensitivity. Results from ANOVA are described in the text (RESULTS section) and the individual *p* values (*post hoc* analysis) are indicated in the graphs. Results were considered statistically significant when *p* < 0.05. All statistical analyses were performed using GraphPad Prism version 9 (GraphPad Software, San Diego, CA, USA) for Windows.

## RESULTS

### Sleep restriction per se does not change the periorbital mechanical threshold of male and female rats

**FIGURE 2:**
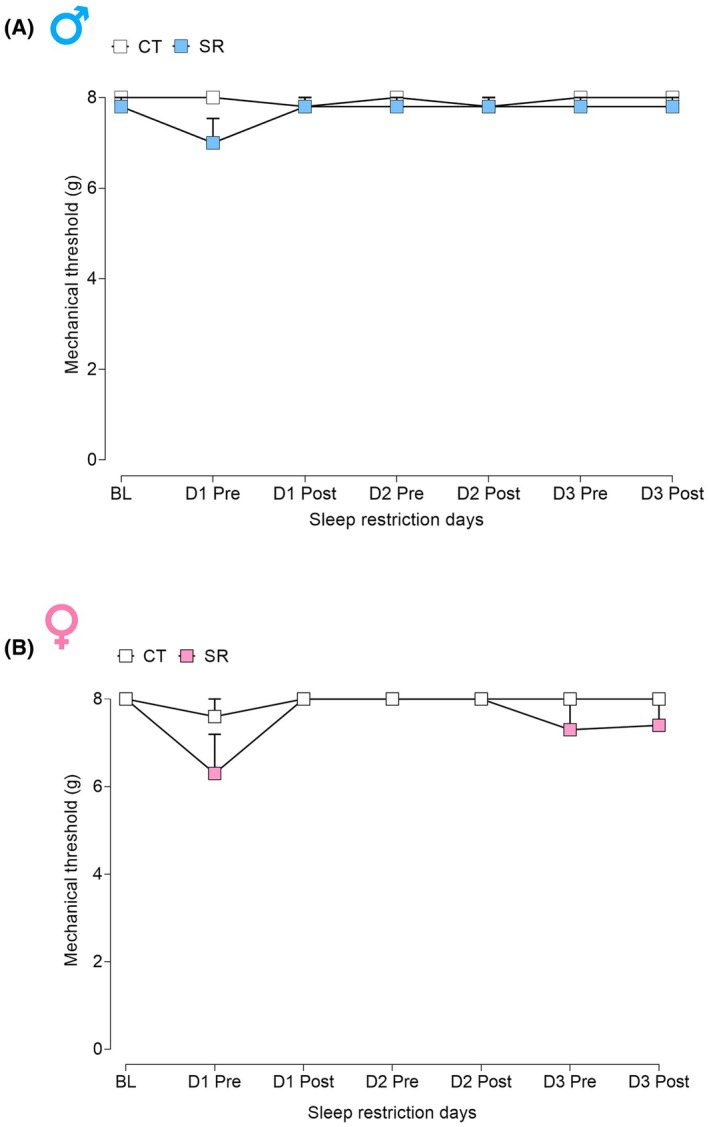
Influence of SR on the periorbital mechanical threshold of male and female rats. The baseline periorbital mechanical threshold was assessed (BL) followed by 3 consecutive days (D1–D3) of sleep restriction. The periorbital mechanical threshold of male (A) and female (B) rats was assessed every day before (Pre) and after (Post) the SR protocol. Data are expressed as mean ± SEM (*n* = 10/group). Two‐way ANOVA with repeated measures followed by Bonferroni *post hoc* test. CT, control; SR, sleep restriction. [Color figure can be viewed at wileyonlinelibrary.com]

Six hours a day of SR for 3 consecutive days did not change the periorbital mechanical threshold in male (Figure 2A, *F*(6,108) = 1.913, *p* = 0.085) and female (Figure 2B, *F*(6,108) = 1.048, *p* = 0.398) rats.

### Sleep restriction sensitizes the trigeminal system to CGRP injected into the TG of female but not male rats

**FIGURE 3:**
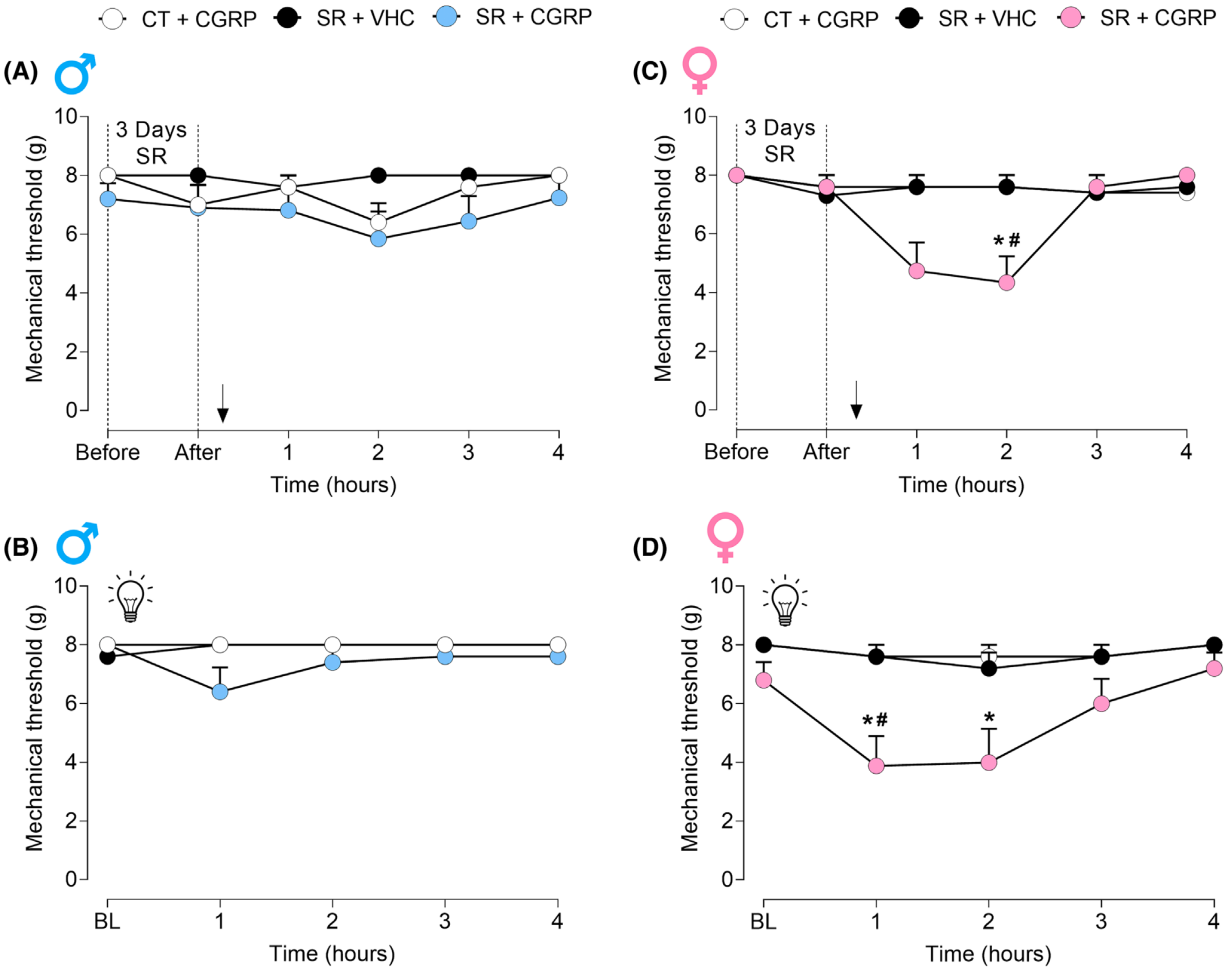
Influence of SR on the effects of CGRP in male and female rats. The baseline periorbital mechanical threshold was assessed (BL), followed by 3 consecutive days of SR. CGRP was injected into the TG at the end of the restriction period (indicated by the arrow). The periorbital mechanical threshold of male (A) and female (C) rats was assessed before (Before) and after (After) the SR, and at 1, 2, 3, and 4 h following CGRP injection. Twenty‐four hours after injection, males (B) and females (D) were exposed to light, and the mechanical threshold was reassessed up to 4 h. Data are expressed as mean ± SEM (*n* = 10/group). *p < 0.05 compared to the CT + CGRP group and ^#^ *p* < 0.05 compared to the SR + VEH group. Two‐way ANOVA with repeated measures followed by Bonferroni *post hoc* test. CGRP, calcitonin gene‐related peptide; CT, control; SR, sleep restriction; VEH, vehicle. [Color figure can be viewed at wileyonlinelibrary.com]

Six hours a day of SR for 3 consecutive days did not change the periorbital mechanical threshold in male rats that received a behaviorally ineffective low dose of CGRP into the TG (Figure 3A, *F*(10,135) = 0.895, *p* = 0.539). Similarly, exposure to an intense, aversive light stimulus, 24 h after the injection, failed to alter periorbital mechanical threshold in males (Figure 3B, *F*(8,108) = 1. 609, *p* = 0.130).

In contrast, the same SR protocol rendered female rats sensitive to that otherwise behaviorally ineffective low dose of CGRP administered into the TG. Periorbital mechanical threshold was significantly decreased 2 h after injection in sleep deprived females (Figure 3C, *F*(10,135) = 6.819, *p* < 0.001). Twenty‐four hours after CGRP injection, exposure to an aversive light stimulus reinstated periorbital mechanical allodynia in female rats (Figure 3D, *F*(8,108) = 2.771, *p* = 0.008), with significant effects at 1 and 2 h post‐exposure.

### Sleep restriction sensitizes the trigeminal system to PACAP injected into the TG of female rats

**FIGURE 4:**
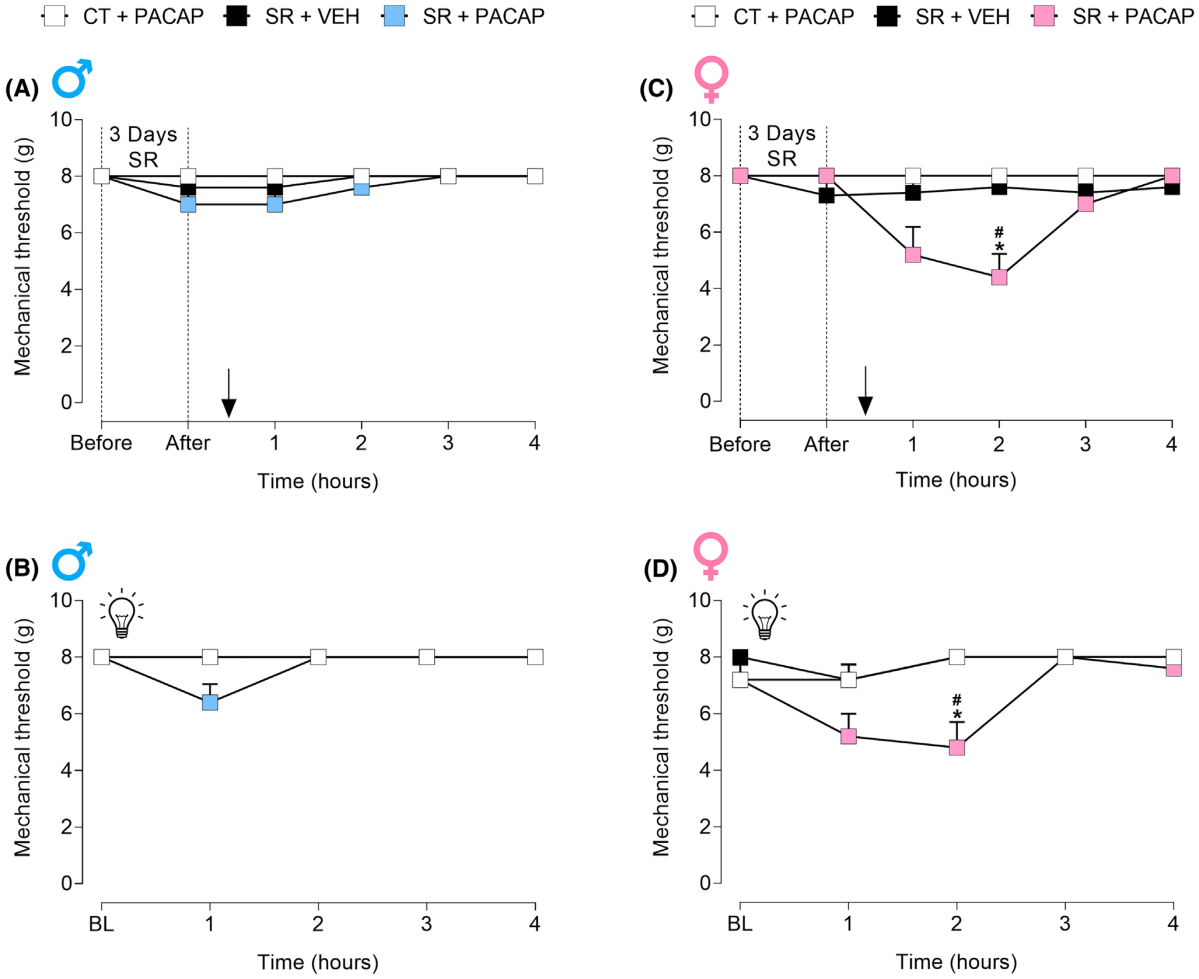
Influence of SR on the effects of PACAP in male and female rats. The baseline periorbital mechanical threshold was assessed (BL), followed by 3 consecutive days of SR. PACAP was injected into the TG at the end of the restriction period (indicated by the arrow). The periorbital mechanical threshold of male (A) and female (C) rats was assessed before and after the SR, and at 1, 2, 3, and 4 h following PACAP injection. Twenty‐four hours after injection, males (B) and females (D) were exposed to light, and the mechanical threshold was reassessed up to 4 h. Data are expressed as mean ± SEM (*n* = 10/group). *p < 0.05 compared to the CT + PACAP group and ^#^ *p* < 0.05 compared to the SR + VEH group. Two‐way ANOVA with repeated measures followed by Bonferroni *post hoc* test. CT, control; PACAP, pituitary adenylate cyclase‐activating polypeptide; SR, sleep restriction; VEH, vehicle. [Color figure can be viewed at wileyonlinelibrary.com]

Three days after SR, intraganglionic injection of PACAP at a low dose did not change the periorbital mechanical threshold in male rats (Figure 4A, *F*(10,135) = 1.061, *p* = 0.397). Similarly, after light exposure, male rats showed no change in their periorbital mechanical threshold (Figure 4B, *F*(8,108) = 6.000, *p* < 0.001).

In females subjected to SR for 3 days, intraganglionic injection of PACAP caused a significant reduction of the periorbital mechanical threshold 2 h after the injection (Figure 4C, *F*(10,135) = 5.846, *p* < 0.001). One day after PACAP injection, the exposure of female rats to an aversive light caused a reactivation of periorbital mechanical allodynia, which was significant at 2 h (Figure 4D, *F*(8,108) = 4.500, *p* < 0.001).

### Caffeine amplifies sex‐dependent susceptibility to migraine‐like responses

**FIGURE 5:**
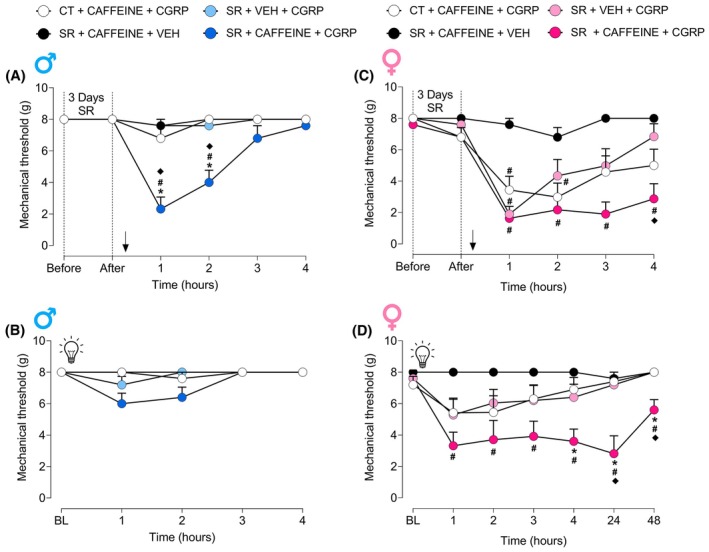
Influence of caffeine on the effects of CGRP in male and female rats subjected to SR. The baseline periorbital mechanical threshold was assessed before 3 consecutive days of caffeine (50 mg/kg) or vehicle (1 mL/kg) oral administration and SR. CGRP was injected into the TG on day 3 (indicated by the arrow). The periorbital mechanical threshold of male (A) and female (C) rats was assessed after the SR, and at 1, 2, 3, and 4 h following CGRP injection. Twenty‐four hours after injection, males (B) and females (D) were exposed to light, and the mechanical threshold was reassessed up to 48 h. Data are expressed as mean ± SEM (*n* = 10/group). *p < 0.05 compared to CT + CAFFEINE + CGRP group, ^#^ *p* < 0.05 compared to SR + CAFFEINE + VEH group, and ^♦^ *p* < 0.05 compared to SR + VEH + CGRP group. Two‐way ANOVA with repeated measures followed by Bonferroni *post hoc* test. CGRP, calcitonin gene‐related peptide; CT, control; SR, sleep restriction; TG, trigeminal ganglion; VEH, vehicle. [Color figure can be viewed at wileyonlinelibrary.com]

When 3 consecutive days of caffeine intake was combined with SR, male rats developed robust periorbital allodynia compared to the non‐SR group at 1 and 2 h after the subthreshold dose of intraganglionic CGRP (Figure 5A, *F*(15,180) = 11.91, *p* < 0.001). Notably, only this triple combination reduced the mechanical periorbital threshold in males, whereas SR alone, SR with subthreshold CGRP, or SR with caffeine (without CGRP) all failed to produce any change. However, even this combination failed to reinstate periorbital allodynia, as males showed no significant change in mechanical threshold after light exposure 24 h later (Figure 5B, *F*(12,144) = 4.102, *p* < 0.001).

In female rats given a subthreshold CGRP dose, 3 consecutive days of caffeine intake triggered migraine‐like symptoms in well‐rested control animals compared to the animals that were subjected to SR and received caffeine, but not CGRP, at 1‐ and 2‐h time points (Figure 5C, *F*(15,180) = 6.047, *p* < 0.001). Moreover, in those subjected to SR, caffeine plus CGRP combination evoked a robust and long‐lasting mechanical allodynia, which was still significant 4 h after injection. At this point, this group differed statistically from the SR groups that receive either CGRP or caffeine. By contrast, caffeine combined with SR had no effect on the periorbital mechanical thresholds in the absence of CGRP at any point evaluated.

One day after CGRP injection, exposure of female rats to the aversive light reinstated periorbital allodynia in a treatment‐dependent manner. The triple combination of caffeine, SR, and subthreshold CGRP produced a robust decrease in the periorbital mechanical threshold that remained significant 48 h after the light exposure (Figure 5D, *F*(18,216) = 2.170, *p* = 0.005). At the 24‐ and 48‐h time points, this group differed statistically from the nonrestricted group treated with caffeine and CGRP, as well as from the SR groups that received either CGRP or caffeine.

### Caffeine enhances PACAP‐induced periorbital allodynia following sleep restriction

**FIGURE 6:**
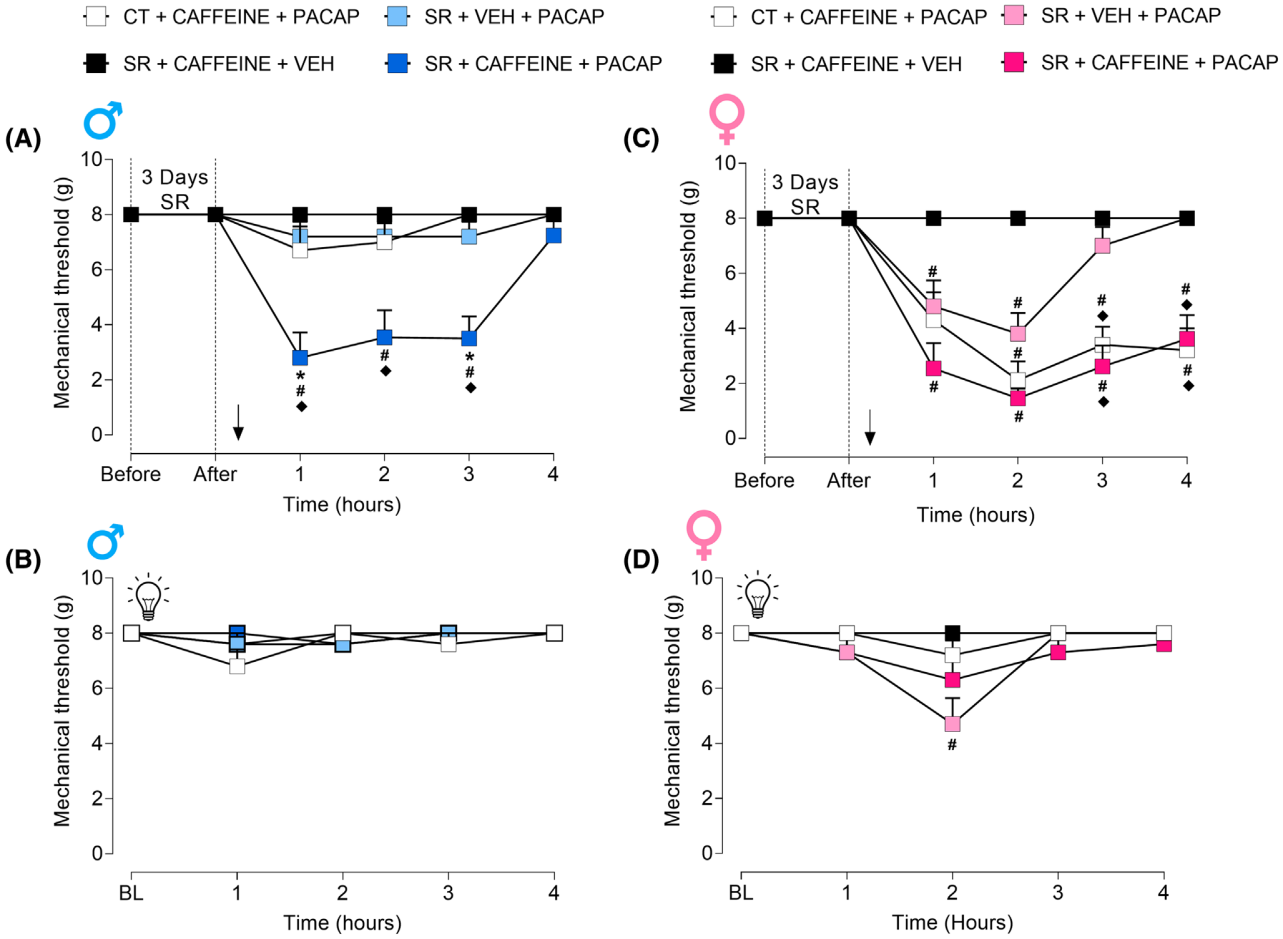
Influence of caffeine on the effects of PACAP in male and female rats subjected to SR. The periorbital mechanical threshold was assessed before 3 consecutive days of caffeine (50 mg/kg) or vehicle (1 mL/kg) oral administration and SR. PACAP was injected into the TG on day 3 (indicated by the arrow). The periorbital mechanical threshold of male (A) and female (C) rats was assessed after the SR, and at 1, 2, 3, and 4 h following PACAP injection. Twenty‐four hours after injection, males (B) and females (D) were exposed to light, and the mechanical threshold was reassessed up to 4 h. Data are expressed as mean ± SEM (*n* = 10/group). *p < 0.05 compared to CT + CAFFEINE + PACAP group, ^#^ *p* < 0.05 compared to SR + CAFFEINE + VEH group, and ^♦^ *p* < 0.05 compared to SR + VEH + PACAP group. Two‐way ANOVA with repeated measures followed by Bonferroni *post hoc* test. CT, control; PACAP, pituitary adenylate cyclase‐activating polypeptide; SR, sleep restriction; TG, trigeminal ganglion; VEH, vehicle. [Color figure can be viewed at wileyonlinelibrary.com]

In male rats, the combination of SR plus caffeine or SR plus PACAP did not change the periorbital mechanical threshold. However, the combination of SR plus caffeine and PACAP induced periorbital mechanical allodynia that was significant from 1 up to 3 h after PACAP injection into the TG (Figure 6A, *F*(15,180) = 7.344, *p* < 0.001). The exposure of male rats to light in the following day had no significant effect on the periorbital mechanical threshold (Figure 6B, *F*(12,144) = 1.262, *p* = 0.247).

In female rats that were subject to SR and received caffeine for 3 consecutive days, the periorbital mechanical threshold was not changed. The combination of SR plus PACAP transitorily decreased the periorbital mechanical threshold compared to the SR group that received caffeine at 1 and 2 h time points (Figure 6C, *F*(15,180) = 10.59, *p* < 0.001). Moreover, the combination of caffeine and a subthreshold dose of PACAP triggered migraine‐like symptoms in well‐rested control animals, as well in the animals subjected to the SR protocol. The responses persisted for at least 4 h for both groups.

One day later, after exposure of female rats to the light, only the SR group that received PACAP showed a reduction in the periorbital mechanical threshold, which was significantly different from the SR group that received only caffeine at the 2‐h time point (Figure 6D, *F*(12,144) = 1.262, *p* = 0.247).

## DISCUSSION

There is a well‐documented bidirectional relationship between migraine and poor sleep, but its underlying mechanisms remain elusive. In previous studies, we modeled migraine in rats by injecting CGRP or PACAP into the TG. In these models, intraganglionic administration produces sumatriptan‐sensitive periorbital allodynia that is subsequently accompanied by light‐evoked photosensitivity, which are more pronounced and longer‐lasting in females.^5^, ^6^ In the present study, we administered behaviorally ineffective (i.e., subthreshold) doses of CGRP or PACAP into the TG to assess whether SR, caffeine intake, or their combination increase susceptibility to migraine‐like responses in both sexes. The main findings indicate that (1) SR for 3 consecutive days (6 h/day) did not alter the periorbital mechanical thresholds in vehicle‐treated rats; (2) SR combined with a subthreshold intraganglionic injection of CGRP or PACAP precipitated migraine‐like responses in females, but not in males, indicating that SR lowers the trigeminovascular activation threshold in females; and (3) 3 days of caffeine intake precipitated migraine‐like responses to subthreshold CGRP or PACAP in non–sleep‐restricted females and exacerbated these responses in sleep‐restricted ones, whereas in males, caffeine combined with SR rendered them susceptible to migraine‐like responses elicited by subthreshold CGRP or PACAP.

Sleep deficit is known to enhance pain sensitivity, although the underlying mechanisms remain poorly understood.^17^, ^19^ In the protocol of SR used herein, the animals were prevented from sleeping during the first 6 h of the light phase, when sleep pressure is highest in rodents.^17^ This procedure has been shown to reduce approximately 98% of slow‐wave sleep and abolishes REM sleep,^17^ but the consequences in the induction of nociceptive responses are variable. Previous studies showed that 6 h a day of total SR over 3 days progressively lowered the mechanical nociceptive threshold in the paw of rats, with no sex differences observed.^19^ Likewise, acute (9–12 h) or repeated (6 h/day for 5 days) SR reduced both thermal and mechanical paw thresholds in mice.^17^ These observations led us to first investigate whether SR would also decrease the periorbital mechanical threshold. According to our results, 6 h of SR for 3 consecutive days did not change the periorbital mechanical threshold in male and female rats. This finding corroborates previous demonstrations that acute SR for 6 h did not change the hind paw or periorbital mechanical thresholds in male and female mice.^10^, ^17^ One possible reason for this difference is the type of mechanical stimulus applied. According to Alexandre et al.,^17^ after SR, only sensitivity to high‐intensity mechanical stimulation increases, whereas responses to nonnoxious mechanical stimuli, such as gentle brush or low intensity von Frey filaments, remain unchanged.

Previous studies of our group have demonstrated that the injection of CGRP into the TG causes periorbital mechanical allodynia and photosensitivity in male and female rodents, but the responses are more pronounced and longer lasting in females.^5^, ^20^ In the present study, a subthreshold dose of CGRP was injected into the TG at the end of the 3‐day SR protocol. The combination of SR and subthreshold CGRP precipitated migraine‐like responses only in females, corroborating previous reports of higher sensitivity of females to CGRP and of female‐selective mechanisms promoting migraine.^5^, ^20^, ^21^, ^22^, ^23^ These findings indicate that SR can act as a priming mechanism to induce a state of vulnerability to the development of migraine‐like responses. When the same animals were exposed to an aversive light for 1 h, 1 day after CGRP injection, the mechanical allodynia was reactivated, indicating long‐lasting trigeminal sensitization. Exposure to aversive light has been widely used to reveal latent sensitization in migraine models.^5^, ^6^, ^20^, ^24^, ^25^ It has been shown that the exposure of sensitized animals to the bright light has been shown to cause c‐Fos activation in neurons of the trigeminal nucleus caudalis, an indication of central sensitization.^25^ In addition, it caused peripheral changes in the trigeminal system including enhanced expression of CGRP and nitric oxide synthase, as well as glial cell in the TG that contribute to enhanced neuron excitability.^26^ Photophobia (i.e., light‐aversion) is a symptom commonly reported in patients with migraine,^27^ and different protocols are used in preclinical settings to evaluate photosensitivity in animals subjected to stimuli that induce migraine‐like responses.^22^ We have previously demonstrated that CGRP injection can induce photosensitivity in male and female rats,^5^ but in the present study the combination of SR plus a subthreshold dose of CGRP caused photosensitivity only in females, suggesting sex differences in the sensitizing effect of SR.

Like CGRP, PACAP is a vasodilatory peptide that can cause migraine‐like attacks when infused into patients and migraine‐like responses when injected into rodents, but mechanistic studies suggest that these peptides largely act independently from one another.^6^, ^28^, ^29^ We have also previously shown that PACAP injection into the TG causes mechanical allodynia and photosensitivity, assessed 24 h after injection, in female, but not in male rats.^6^ When injected systemically, PACAP induced light aversion in male and female mice through CGRP‐independent mechanisms.^28^ In the present study, a subthreshold dose of PACAP was administered into the TG at the end of the 3‐day SR protocol, leading to an outcome comparable to that of the CGRP injection. Only female rats developed significant transitory periorbital mechanical allodynia, which was reactivated 24 h later by aversive light exposure. To our knowledge, this is the first study to evaluate how SR interacts with PACAP to induce migraine‐like symptoms, showing that SR sensitizes the trigeminovascular system to subthreshold PACAP and selectively elicits migraine‐like responses in females.

Few preclinical and clinical studies have explored how sleep loss contributes to migraine development in susceptible individuals. Although these studies relied on short (6–12 h) SR protocols, their findings are largely consistent with our results. Acute SR followed by a subthreshold dose of either systemic NTG or supradural CGRP induced periorbital and hind paw mechanical allodynia in female mice, which was observed at the end of the 6‐h SR period and became more pronounced 24 h later.^10^ Interestingly, when the migraine provoking stimulus was administered before SR, sleep parameters remained unchanged, indicating that migraine‐like pain does not significantly disrupt sleep.^10^ Additionally, acute SR (6 or 12 h) significantly increased the frequency of cortical spreading depression induced by application of KCl to the pial surface.^30^ Human transcranial magnetic stimulation studies indicate that insufficient sleep perturbs cortical inhibitory/excitatory balance in patients with migraine,^31^, ^32^ consistent with a state of heightened cortical responsivity that could facilitate cortical spreading depression (and thus aura) and promote attack initiation in sleep‐deprived individuals. Taken together these results provide convergent evidence that SR, whether acute (6–12 h) or prolonged (3 days), markedly increases susceptibility to migraine. However, our study is the only to directly contrast male and female responses under the same SR protocol and it found that SR‐induced susceptibility occurred exclusively in females. It therefore remains unresolved whether the absence of effect in males reflects true resistance or merely a higher sensitivity threshold. Addressing this question will require systematic dose–response experiments that escalate CGRP and PACAP doses. It is important to mention that differential sensitivity of males and females to CGRP have been extensively reported in the literature, and more recently sex differences have also been pointed out for PACAP effects.^22^, ^23^, ^33^ Selective female and male mechanisms related to migraine have been described^23^, ^34^ but have not been explored in the context of SR. Therefore, it is quite likely that biological differences between the sexes contribute to the outcome caused by SR in males and females, but this issue still needs to be investigated.

Caffeine is one of the most widely consumed psychoactive substances worldwide^35^ and the consumption tends to be higher among individuals with poor sleep.^36^ Habitual or high‐dose intake has been associated with increased headache frequency and severity.^12^, ^37^ Despite these interconnections, no studies to date have examined the combined effects of caffeine intake, poor sleep, and migraine‐related outcomes across human or animal subjects. Our data indicate that caffeine lowers the trigeminovascular activation threshold for migraine‐like symptoms. The dose of caffeine used herein has been shown to produce stable plasmatic levels (25 μg/mL) up to 4 h.^15^ As the behavioral responses were evaluated at least 6 h after caffeine administration or in the absence of the treatment (day 4), it is unlikely that caffeine causes a direct effect, but it somehow modulates the sensitivity of the trigeminal neurons favoring a pronociceptive state. Females were markedly more susceptible: 3 days of caffeine intake precipitated migraine‐like responses in non–sleep‐restricted female rats receiving subthreshold doses of CGRP or PACAP into the TG. These findings suggest that, in susceptible females, caffeine consumption alone can act as a risk factor for developing migraine. When caffeine intake is combined with SR, their effects are synergic, further increasing susceptibility in females and leading to significantly exacerbated migraine‐like responses. Notably, the combination of SR and caffeine intake uniquely renders male rats susceptible to migraine‐like responses triggered by subthreshold CGRP or PACAP, but this effect is transitory, since it was not reactivated by aversive light exposure. These findings underscore the role of caffeine as a modulator of trigeminovascular sensitivity, highlighting a pronounced sex‐dependent effect. Although plasma caffeine is rapidly cleared, it has been demonstrated that caffeine exposure can produce persistent alterations in the neural environment that may influence how the brain responds to subsequent challenges.^38^, ^39^ Therefore, early caffeine exposure can modulate the neural milieu in a way that amplifies the impact of subsequent SR. Caffeine exerts an ambivalent role in migraine: it can enhance acute analgesic responses yet act as a trigger or withdrawal precipitant in susceptible individuals.^12^, ^13^ Mechanistically, caffeine is a potent antagonist of adenosine receptors with roughly equally high affinity for both A_1_ and A_2A_ receptors, which are widely expressed in the trigeminovascular system.^40^ Several studies have reported antinociceptive effects of adenosine that are likely to be mediated by A_1_ receptors.^41^ In migraine models, activation of A_1_ receptors can inhibit neurogenic vasodilation and CGRP release, whereas A_2_ receptors activation results in dural vasodilation, but does not modulate trigeminal CGRP release.^16^, ^42^, ^43^ Thus, it is possible that caffeine‐induced blockade of A_1_ receptors within the trigeminovascular system impairs the inhibitory influence driven by A_1_ receptor activation, thereby potentializing the pronociceptive effects of CGRP.

Collectively, our data demonstrate that poor sleep and caffeine intake, individually and particularly in combination, are key determinants of migraine susceptibility, with females being markedly more sensitive. The findings highlight the importance of considering lifestyle factors in migraine risk assessment and prevention, and provide a translational framework for understanding how environmental and behavioral triggers interact with trigeminovascular pathways. Future studies should investigate the underlying mechanisms of these sex‐specific responses and evaluate whether interventions targeting sleep quality and caffeine consumption can mitigate migraine risk in susceptible populations.

Although our findings provide valuable insights into the interaction between SR, caffeine intake, and migraine susceptibility, some limitations should be noted. First, as with all animal studies, species‐specific differences in physiology and hormonal regulation may limit the direct translation of these results to humans. Second, migraine‐like responses were inferred from specific behavioral end points, which may not fully capture the complete spectrum of migraine phenotypes, highlighting an inherent limitation of preclinical models. Finally, although clear sex‐specific differences in susceptibility were observed, the precise hormonal, neuronal, and molecular mechanisms underlying these effects remain to be elucidated, warranting further mechanistic investigation.

## AUTHOR CONTRIBUTIONS

**Gabriel Camargo de Oliveira:** Investigation; writing – review and editing; methodology; formal analysis. **Darciane Favero Baggio:** Investigation; methodology; writing – review and editing; formal analysis. **Vanessa Bordenowsky Pereira Lejeune:** Investigation; writing – review and editing. **Laura Storithont Quinelato:** Investigation; writing – review and editing. **Fernanda Mariano Ribeiro da Luz:** Investigation; writing – review and editing. **Aleksander Roberto Zampronio:** Conceptualization; writing – review and editing; supervision. **Luana Fischer:** Conceptualization; writing – original draft; writing – review and editing; supervision. **Juliana Geremias Chichorro:** Conceptualization; funding acquisition; writing – original draft; writing – review and editing; supervision.

## FUNDING INFORMATION

This work was supported by Coordenação de Aperfeiçoamento de Pessoal de Nível Superior (CAPES, Brazil, financial code 001), Conselho Nacional de Desenvolvimento Científico e Tecnológico (CNPq, Brazil), and Fundação Araucária de Apoio ao Desenvolvimento Científico e Tecnológico do Estado do Paraná (call 04‐2023, grant 3527). Gabriel Camargo de Oliveira, Darciane Favero Baggio and Vanessa Bordenowsky Pereira Lejeune are recipients of scholarships provided by CAPES. Juliana Geremias Chichorro and Aleksander Roberto Zampronio are recipients of Research Productivity Scholarships from CNPq.

## CONFLICT OF INTEREST STATEMENT

**Gabriel Camargo de Oliveira, Darciane Favero Baggio, Vanessa Bordenowsky Pereira Lejeune, Laura Storithont Quinelato, Fernanda Mariano Ribeiro da Luz, Aleksander Roberto Zampronio, Luana Fischer**, and **Juliana Geremias Chichorro** declare no conflicts of interest related to this work.
